# 

**Published:** 1886-08

**Authors:** 


### HALL'S JOURNAL OF HEALTH

Vol 33. AUGUST, 1886. No. 8

### *SPECIAL NOTICE.*

The business headquarters of Hall's Journal of Health have been removed to new and commodious offices at 206 Broadway, Evening Post Building, entrance Room 11, one of the most central locations in the city of New York, to which all communications and "exchanges" should be hereafter addressed, and where all local business relating to the Journal will be transacted.

Journal of Health Publishing Co.,
206 Broadway.

### *HALL'S JOURNAL OF HEALTH*

Is now completing the THIRTY-THIRD YEAR of its continuous publication, during which period it has never missed a single monthly issue. Its patrons are distributed over every State and Territory of our broad domain, with no inconsiderable number in England and the British Provinces.

It is, and will continue to be, wholly independent of any particular school, dealing only with *facts*, in science, in medicine and in art. Its "Miscellaneous" columns are meant to be instructive and diverting, and its "Home Department" replete with information of value to every household.

Every number of the JOURNAL, it is believed, will be worth to the subscriber the cost of a year's subscription, and there is no reason why under its present liberal management, its circulation should not be doubled within the present year. The present indications are that it will even exceed this.

*To advertisers* the JOURNAL offers in some respects unequalled advantages, and should receive no lack of patronage from such as desire to enlarge their field of enterprise.

Price of yearly subscription, $1.

We have a limited number of back numbers wherewith to fill orders dating from January, and such as would have them should apply in season. Address in all cases

JOURNAL OF HEALTH PUBLISHING CO.,
206 Broadway, New York City.

